# Patient and health facility attributes associated with retention and virologic suppression in private for-profit health facilities in Nigeria

**DOI:** 10.1186/s12981-022-00438-3

**Published:** 2022-02-22

**Authors:** Muyi Aina, Zeena Yesufu, Abdulateef Salisu, Echezona Ezeanolue, Charles Mensah, Patrick Dakum

**Affiliations:** 1 Solina Center for International Development and Research (SCIDaR), 8, Libreville Street, Wuse 2, Abuja, Nigeria; 2 Healthy Sunrise Foundation, Las Vegas, USA; 3 Institute of Human Virology, Nigeria (IHVN), Abuja, Nigeria

**Keywords:** HIV, AIDS, Private facilities, Implementation science, Nigeria, Patient outcomes

## Abstract

**Background:**

In Nigeria, private for-profit health facilities present an opportunity to achieve the UNAIDS 95-95-95 HIV targets because of their reach and patronage. However, little is known about determinants of outcomes in these facilities. This study describes patient outcomes and the patient and health facility characteristics associated with these outcomes in adults receiving HIV treatment in private facilities in the Federal Capital Territory (FCT), Benue and Nasarawa states in north-central Nigeria.

**Methods:**

A retrospective longitudinal analysis of program data collected between 2013 and 2019 was done. Patient attributes and outcomes were compared across the two states and FCT. Incidence rates were determined for all cause exit, mortality and loss to follow up (LTFU). Cox proportional hazard models were used to identify associations between patient and facility attributes and these outcomes. Bivariate and multivariate logistic regression models were used to determine the factors associated with viral suppression among the study participants.

**Results:**

Of the 22,010 study subjects, 42.7%, 22.2% and 35.1%, respectively, were in Benue, FCT and Nasarawa. Almost a third (31.8%) had received antiretroviral treatment (ART) for less than a year at censoring. Incidence rates for all-cause exit, mortality and loss to follow up (LTFU) were 17.2 (95% CI 16.8, 17.5), 2.1 (95% CI 2.0, 2.2), and 11.2 (95% CI 10.8, 11.8) per 100 person years respectively. Males had higher risks of death (HR = 1.47, 95% CI 1.25–1.73), and LTFU (HR = 1.08, 95% CI 1.00–1.16). Age at ART start showed a dose–response association with both mortality and LTFU. Care at model facilities (OR = 2.16, *p* < *0.001*), Zidovudine (AZT)-based regimens (OR = 2.00, *p* < *0.001*), and lowest quartile baseline CD4 + count (OR = 2.40, *p* < *0.001*) were associated with regimen switch. 75.6% of subjects were viral suppressed. Male gender (OR = 0.84, *p* = *0.025*); AZT-based regimen (OR = 0.72, *p* < *0.001*), age in the bottom quartile (OR = 0.71, *p* = *0.002*) were associated with virally suppression.

**Conclusion:**

Private for-profit facilities are a major provider of HIV and other health services in Nigeria. With appropriate technical support and engagement, they can help accelerate efforts to achieve epidemic control of HIV in Nigeria, and contribute to achievement of UNAIDS 95-95-95 target by 2030.

## Introduction

The 2018 Nigerian National AIDS Indicator and Impact Survey (NAIIS), the largest ever population-based HIV impact assessment (PHIA) globally, showed a prevalence of 1.4% among people aged 15–69 years [1]. This represents a remarkable decline in HIV disease burden since the country began using sentinel surveys to track its epidemic. Prior to this, the national prevalence rose from 1.8% in 1991 to a peak of 5.8% in 2001 before declining to 5.0% in 2003 and 2.3% in 2015 [2–5]. This trend suggests that the massive efforts of the government and its development and donor partners to control the HIV epidemic during the last three decades have moved the country closer to achieving epidemic control [3, 6–10].

With its estimated 1.9 million people living with HIV, Nigeria remains the 4^th^ largest contributor to the global burden of HIV [1]. In 2017, the country accounted solely for more than half of the 260,000 new HIV infections in West and Central Africa, of which 36,000 were in children [11]. It also contributed over half of deaths due to AIDS related illnesses in the region [11]. Nigeria’s HIV epidemic is mixed, with persisting pockets of high prevalence [12]. The national HIV prevalence of 1.4% masks subnational variations that range from 0.3% in Kebbi, Katsina and Jigawa States in the northwest region, to 5.5% in Akwa-Ibom state in the south-south region [1, 6]. Only 7 out of the 36 states in the country account for more than 50% of the national HIV burden, and 80% of the combined burden is attributable to only 13 of the 37 states [1, 13]. These disparities reflect wide inequities in poverty, education and access to HIV prevention services, as well as geographical differences in high-risk behaviors and harmful socio-cultural practices, stigma and discrimination [2, 12, 14].

The HIV response in Nigeria is government-led but largely donor-funded, with many programs initially focusing on supporting the public sector’s capacity to provide comprehensive HIV prevention, care and treatment services in secondary and tertiary health facilities [7, 8, 15, 16]. However, coverage of services remained limited by the inadequate number of service delivery points [17]. To bridge the coverage gaps, the government and its partners rolled out a strategy to decentralize ART to primary health centers and implemented a task shifting policy that allowed nurses and allied health workers to be trained and empowered to provide varying levels of services in the HIV prevention, care and treatment continuum [18–20]. Nigeria is yet to achieve the UNAIDS 90-90-90 targets set for 2020, with only about 1 million people, approximately 52% of people living with HIV (PLHIV), currently on ART [11, 21, 22]. Despite this huge gap in service coverage, the potential for Nigeria to expand access to HIV services through private providers remains largely under-tapped [20, 23]. Up to 65% of all citizens, and 72% of the poorest quintile, access basic health services from private sector outlets [24, 25]. As such, private providers can not only help accelerate progress towards the revised 95-95-95 targets by 2030, but also reduce inequities in access to HIV prevention, care and treatment services. There is currently little or no data on performance of private facilities in provision of HIV services in Nigeria.

Solina Center for International Development and Research (SCIDaR), with sub-grant funding from the Institute of Human Virology, Nigeria (IHVN), designed and began implementing a model to support private for-profit facilities to provide HIV prevention, care and treatment services in Nigeria in 2013. This paper aims to determine the patient and health facility factors that are associated with patient retention and virologic suppression in adult patients receiving ART in private health facilities supported by the SCIDaR-IHVN program in Benue and Nasarawa states, and the Federal Capital Territory (FCT) between 2013 and 2019. Benue, Nasarawa and the FCT are all in the north-central zone of the country, with HIV prevalence rates of 5.3%, 2.0% and 1.6% respectively, compared to a national average of 1.4% [1]. The CDC/PEPFAR-funded HIV program covers most direct HIV care costs, including antiretroviral medicines and laboratory tests (CD4 testing, viral load, resistance testing). However, some private facilities charge an out-of-pocket consultation fee for patients.

## Methods

This study is a retrospective analysis of routinely collected longitudinal data for patients enrolled in the SCIDaR-IHVN private sector HIV care and treatment program in Benue, Nasarawa, and the FCT between January 2013 and November 2019. All adult patients (aged 18 years and above), who received ART for at least one month in any of the 221 private health facilities supported by the program (81 in Benue, 75 in FCT, and 65 in Nasarawa) were included in this study. Patients on ART for less than a month were excluded as they are unlikely to have demonstrated treatment effects. Also, because medication usage was not directly observed in most patients, only those who returned for the first follow up visit at one month could be presumed to have commenced treatment. Routinely collected patient-level demographic and clinical data was extracted from the PEPFAR Retention and Audit Determination Tool (RADET) database. The data was cleaned, organized and analyzed using Stata® 13 SE [26].

### Data analysis

The outcomes of interest were viral suppression (< 1000 copies/ml) in most recent viral load test [27], all-cause exit, death, loss to follow up (LTFU, defined as patients who fail to return for drug refills for 3 months), and regimen switch to 2nd line. Demographic and clinical care characteristics were compared across states using Pearson’s chi squared tests of proportions for categorical variables, including gender, pregnancy status, year enrolled in ART, duration on ART (years), starting ART regimen base (Tenofovir, Zidovudine or other), current ART treatment line (1st or 2nd line), ART refill appointment duration (1, 2 and 3 or more months), health facility location (rural versus urban) and host health facility support type (model facilities had higher numbers of enrolled patients on the program and received more intensive technical support in form of lower facility to Quality Improvement Team (QIT) staff ratios compared to standard support facilities). Means of continuous variables (age at ART start, baseline CD4+ count) were compared across states with one-way ANOVA.

Treating time on ART, in months, at point of exit as the analysis time, overall and state-specific incidence rates with 95% confidence intervals were calculated for all-cause exit, mortality, LTFU, treatment stoppage and transfers out. In addition, state-specific and overall incidence rates of regimen switch from 1st to 2nd line were determined. Hazard ratios (with 95% confidence intervals) were computed using Cox proportional hazards models to identify demographic, clinical and patient care characteristics that were associated with all-cause exit, mortality, and LTFU in the study population. In addition, odds ratios, with 95% confidence intervals) were computed with a logistic regression model to identify patient characteristics that were associated with regimen switch to 2nd line.

Viral suppression rates (proportion of patients with most recent viral load < 1000 c/ml) were calculated for each state and for the overall study population. The state-specific rates were compared using a Pearson chi squared test. Factors associated with viral suppression in the study population were determined using multivariate logistic regression models. Patient characteristics assessed for relationship with viral suppression included gender, age at ART start (years, categorized into quartiles), state of residence, pregnancy status, host health facility support type, ART enrolment year, duration on ART in years, months of ARV refill (1 month, 2 months, or ≥ 3 months), baseline CD4+ count (categorized into quartiles), current regimen base, and contact testing status. Adjusted ORs for viral suppression by ART enrolment year and by duration on ART in years from the best fit multivariate logistic regression model were charted to identify any trends over time.

## Results

**Fig. 1:**
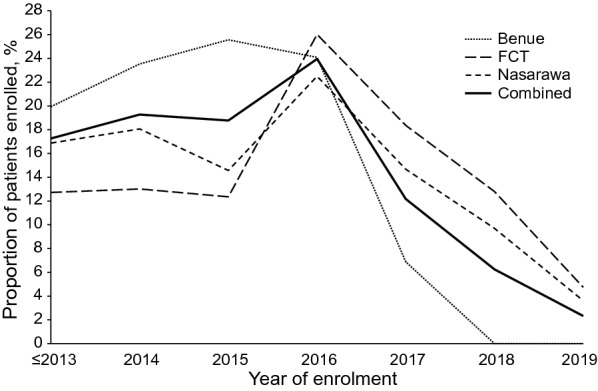
Trends in patient enrolment by year

A total of 22010 patients meeting the inclusion criteria were included in this analysis. This number included 9392 participants (42.7%) in Benue, 4890 (22.2%) in the FCT, and 7728 (35.1%) in Nasarawa. The means and standard deviations of patient numbers per facility were 116 (SD 219.8) in Benue, 65.2 (SD 60.4) in FCT, and 118.9 (90.6) in Nasarawa. Two percent (442) of patients had commenced ART prior to joining the program in 2013. As shown in Table 1, majority (70.9%) were female, with a significantly higher proportion of females in Benue than FCT and Nasarawa (*p* < *0.001*). Among the female study subjects, 3.9% were pregnant and 3.6% breastfeeding at time of data recording. Mean age at start of ART was 33.8 years (SD 9.9 years). Overall, almost 1 in 6 (17.3%) patients commenced ART in 2013 or earlier, with Benue contributing more to this group (19.9%) compared to FCT (12.7%) or Nasarawa (16.9%). Annual enrolment increased gradually to peak at 24.0% in 2016, after which enrolment began to decline as shown in Fig. 1. As at time of exit from the program or censoring, 31.8% of subjects had been on ART for a year or less and 12.9% had received ART for 4 years or more. Mean baseline CD4 + count was slightly higher for Benue-based patients at 385.2 cells/ml, compared to the overall mean of 374.5 cells/ml (*p* < *0.001*). The majority (81.5%) of patients were started on tenofovir (TDF)-based treatment regimens and few (1.0%) had switched to second line regimens as of time of analysis. As Table 1 shows, majority of patients in FCT (54.7%) and Nasarawa (52.4%) refilled their medications monthly, while most patients in Benue (50.7%) refilled every two months. Overall contact testing rate was 85.9% on the program. A slightly lower proportion of clients in Benue (78.0%) had their contacts tested. 44.5% of patients in FCT, 33.7% in Nasarawa and none in Benue received their care in health facilities categorized as model sites.

**Table 1:** Characteristics of study participants

	Benue	FCT	Nasarawa	Combined	p-value
*Sex, N (%)*					
Female	6977 (74.3)	3298 (67.4)	5325 (68.9)	15,600 (70.9)	< 0.001^*^
Male	2415 (25.7)	1597 (32.6)	2403 (31.1)	6410 (29.1)	
*Age at ART start, mean (SD)*	33.8 (10.4)	34.3 (9.4)	33.5 (9.5)	33.8 (9.9)	< 0.001^**^
*Pregnancy status*^†^*, N (%)*					
Not pregnant	6353 (91.1)	3069 (93.1)	5010 (94.1)	14,432 (92.5)	< 0.001^*^
Pregnant	318 (4.6)	116 (3.5)	167 (3.1)	601 (3.9)	
Breastfeeding	306 (4.4)	113 (3.4)	148 (2.8)	567 (3.6)	
*Duration on ART, N (%)*					
0–1 year	3452 (36.8)	1562 (31.9)	1993 (25.8)	7007 (31.8)	< 0.001^*^
1–2 years	2831 (30.1)	1069 (21.9)	1505 (19.5)	5405 (24.6)	
2–3 years	1782 (19.0)	940 (19.2)	1458 (18.9)	4180 (19.0)	
3–4 years	882 (9.4)	583 (11.9)	1118 (14.5)	2583 (11.7)	
4 + years	445 (4.5)	736 (15.1)	1654 (21.4)	2835 (12.9)	
*Baseline CD4* + *count,* *mean (SD)*	385.5 (234.3)	374.5 (283.2)	362.1 (278.0)	374.5 (265.2)	< 0.001^**^
*ART regimen at start, N (%)*					
TDF based	8020 (85.4)	3634 (74.3)	6289 (81.4)	17,943 (81.5)	< 0.001^*^
AZT based	1364 (14.5)	1241 (25.4)	1431 (18.5)	4036 (18.3)	
Other	8 (0.1)	15 (0.3)	8 (0.1)	31 (0.1)	
*Current ART line, N (%)*					
1^st^ line	9345 (99.5)	4811 (98.4)	7625 (98.7)	21,781 (99.0)	< 0.001^*^
2^nd^ line	47 (0.5)	79 (1.6)	103 (1.3)	229 (1.0)	
*ARV refill duration, N (%)*					
1 month	3489 (37.2)	2674 (54.7)	4049 (52.4)	10,212 (46.4)	< 0.001^*^
2 months	4762 (50.7)	1568 (32.1)	2987 (38.7)	9317 (42.3)	
3 + months	1141 (12.2)	648 (13.3)	692 (9.0)	2481 (11.3)	
*Contact tested, N (%)*					
No	174 (78.0)	3649 (83.5)	6230 (87.6)	10,052 (85.9)	< 0.001^*^
Yes	49 (22)	719 (16.5)	882 (12.4)	1650 (14.1)	
*Facility support, N (%)*					
Standard	9392 (100)	2712 (55.5)	5122 (66.3)	17,226 (78.3)	< 0.001^*^
Model^§^	0	2178 (44.5)	2606 (33.7)	4784 (21.7)	
Total	9392 (42.7)	4890 (22.2)	7728 (35.1)	22010 (100)	< 0.001^*^

^†^ Female patients only included in analysis

^‡^ Benue state ceded out of program to another implementer in 2018

^§^ Model facility support approach introduced in 2018 after sites in Benue had been ceded

^*^ P-value from Pearson’s Chi-squared test of proportions

^**^ P-value from one-way ANOVA

### All-cause exit, mortality, LTFU and treatment stoppage

Table 2 shows the crude incidence rates, with 95% CIs, for all-cause exit, mortality, LTFU and treatment stoppage in the study. Overall rate for all-cause exit was 17.2 (95% CI 16.8, 17.5) per 100 person years, with Benue recording 11.3 (95% CI 10.8, 11.8) per 100 person years compared to FCT’s 22.6 (95% CI 21.7, 23.5) and Nasarawa’s 18.9 (95% CI 18.3, 19.5) per 100 person years. Also, the program recorded 2.1 (95% CI 2.0, 2.2) deaths, 11.2 (95% CI 10.9, 11.5) losses to follow up, 0.6 (95% CI 0.6, 0.7) treatment stoppages and 3.2 (95% CI 3.1, 3.4) out-transfers per 100 person years combined. Again, rates for all types of exit were lowest in Benue (Table 2).

**Table 2:** Incidence rates of all-cause exit, mortality, LTFU, treatment stoppage, transfers out and regimen switch

	Incidence rates^†^ (95% CI)			
	Benue	FCT	Nasarawa	Combined
All-cause exit	11.3 (10.8, 11.8)	22.6 (21.7, 23.5)	18.9 (18.3, 19.5)	17.2 (16.8, 17.5)
Mortality	1.8 (1.6, 2.0)	2.3 (2.0, 2.6)	2.3 (2.1, 2.5)	2.1 (2.0, 2.2)
LTFU	7.4 (7.0, 7.9)	15.2 (14.5, 16.0)	12.1 (11.6, 12.6)	11.2 (10.9, 11.5)
Treatment stoppage	0.4 (0.3, 0.5)	0.9 (0.7, 1.1)	0.7 (0.6, 0.8)	0.6 (0.6, 0.7)
Transferred out	1.7 (1.5, 1.9)	4.3 (3.9, 4.7)	3.9 (3.6, 4.2)	3.2 (3.1, 3.4)
Regimen switch to 2nd line	0.3 (0.2, 0.4)	0.5 (0.4, 0.7)	0.5 (0.4, 0.6)	0.4 (0.4, 0.5)

^†^ Incidence rates calculated per 100 person years

FCT – Federal Capital Territory; LTFU – Lost to follow up

Results of multivariate Cox proportional hazard models showing associations of patient attributes with the different types of program exit are shown in Table 3. Overall, males had statistically significant higher risks of all-cause exit (HR 1.10, 95% CI 1.04–1.17), death (HR 1.47, 95% CI 1.25–1.73), and LTFU (HR 1.08, 95% CI 1.00–1.16) than females. Risk of all-cause exit was higher among subjects in FCT (HR 2.61, 95% CI 1.87–3.65) and in Nasarawa (HR 1.97, 95% CI 1.41–2.75) than for subjects in Benue. Likewise, subjects receiving care in facilities located in FCT and Nasarawa had significantly higher risks of being lost to follow up than those in Benue. However, there were no statistically significant differences in risk of mortality across states. Breastfeeding was associated with lower risks of all-cause exit (HR 0.64, 95% CI 0.50–0.81) and LTFU (HR 0.62, 95% CI 0.46–0.83) compared to adults who are neither pregnant nor breastfeeding. Pregnancy was not significantly associated with risk of program exit in this study. As shown in Table 3, subjects’ age at ART start showed a dose–response association with both mortality (lower age progressively associated with decreasing risk of mortality) and LTFU (lower age progressively associated with increasing risk of LTFU). Patients who refilled their ART medications monthly had higher risks of all-cause exit (HR 2.21, 95% CI 2.01–2.43), mortality (HR 3.34, 95% CI 2.40–4.62), and LTFU (HR 2.21, 95% CI 1.97–2.47) than those who refilled quarterly. Contact testing was associated with significantly lower risks of all-cause exit (HR 0.40, 95% CI 0.36–0.44), mortality (HR 0.42, 95% CI 0.31–0.56), and LTFU (HR 0.37, 95% CI 0.32–0.42) in the study population; and those patients receiving their care in model facilities had significantly lower risks of all-cause exit (HR 0.86, 95% CI 0.81–0.91) and LTFU (HR 0.78, 95% CI 0.73–0.83) than those accessing ART in standard support private for-profit facilities.

**Table 3:** Associations between patient characteristics and all-cause exit, mortality and LTFU

	Hazard ratios (95% CI)*		
	All-cause exit	Mortality	LTFU
*Sex*			
Female	1.0	1.0	1.0
Male	1.10 (1.04, 1.17)	1.47 (1.25, 1.73)	1.08 (1.00, 1.16)
*State*			
Benue	1.0	1.0	1.0
FCT	2.61 (1.87, 3.65)	0.99 (0.48, 2.01)	5.22 (2.96, 9.22)
Nasarawa	1.97 (1.41, 2.75)	0.94 (0.45, 1.91)	3.66 (2.08, 6.46)
*Pregnancy status*			
Not pregnant	1.0	1.0	1.0
Pregnant	1.09 (0.92, 1.30)	0.94 (0.48, 2.01)	1.08 (0.87, 1.33)
Breastfeeding	0.64 (0.50, 0.81)	0.32 (0.10, 1.01)	0.62 (0.46, 0.83)
*Age at ART start*^†^			
1st quartile	1.0	1.0	1.0
2nd quartile	0.98 (0.91, 1.06)	0.69 (0.57, 0.84)	1.07 (0.97, 1.18)
3rd quartile	0.10 (0.92, 1.08)	0.62 (0.50, 0.77)	1.12 (1.01, 1.24)
4th quartile	1.16 (1.07, 1.26)	0.54 (0.42, 0.68)	1.38 (1.25, 1.53)
*ARV refill duration*			
3 months	1.0	1.0	1.0
2 months	1.13 (1.02, 1.24)	1.40 (0.99, 1.98)	1.03 (0.91, 1.17)
1 month	2.21 (2.01, 2.43)	3.34 (2.40, 4.62)	2.21 (1.97, 2.49)
*Contact tested*			
No	1.0	1.0	1.0
Yes	0.40 (0.36, 0.44)	0.42 (0.31, 0.56)	0.37 (0.32, 0.42)
*Facility support model*			
Standard	1.0	1.0	1.0
Model	0.86 (0.81, 0.91)	1.12 (0.96, 1.31)	0.78 (0.73, 0.83)

^*^Estimates adjusted for gender, state, year of ART enrollment, pregnancy status, age at start of ART, ART refill appointment duration, ART regimen at start of treatment, baseline CD4, contact testing and type of facility support

^†^ 1st quartile ≥ 41 years; 2nd quartile 33—40 years; 3rd quartile 28—32 years; 4th quartile 18—27 years

LTFU – Lost to follow up

### Regimen switch to 2nd line

The results of unadjusted and adjusted logistic regression modeling of factors associated with regimen switch to 2^nd^ line are presented in Table 4. Patients were significantly more likely to switch if they received care at model facilities (OR 2.17, 95% CI 1.55–3.03); were started on AZT-based regimens versus TDF-based regimens (OR 2.00, 95% CI 1.40–2.85); or had a baseline CD4 + cell count in the bottom quartile versus top quartile (OR 2.40, 95% CI 1.47—3.90). Also, as shown in Table 4, odds of regimen switch increased progressively as time on ART increased up till 3–4 years. Odds of switch were not statistically different between those patients treated for 4 years or more when compared to those treated for less than one year.

**Table 4:** Factors associated with ART regimen switch to second line

Characteristics	Bivariate odds ratio (95% CI)	Multivariate odds ratio (95% CI)
*Facility support type*		
Standard	1.0	1.0
Model	2.45 (1.84, 3.25)	2.17 (1.55, 3.03)
*ART regimen at start*		
TDF based	1.0	1.0
AZT based	2.35 (1.75, 3.16)	2.00 (1.40, 2.85)
Other	4.57 (0.62, 33.74)	4.70 (0.58, 38.01)
*Baseline CD4 (cells/microlitre)*^†^		
Top quartile	1.0	1.0
2nd quartile	1.62 (0.96, 2.73)	1.77 (1.04, 2.99)
3rd quartile	1.22 (0.70, 2.13)	1.21 (0.69, 2.11)
4th quartile	2.54 (1.56, 4.12)	2.40 (1.47, 3.90)
*Duration on ART*		
0–1 year	1.0	1.0
1–2 years	5.03 (1.46, 17.0)	3.27 (0.70, 15.21)
2–3 years	7.60 (2.28, 25.37)	6.87 (1.61, 29.27)
3–4 years	19.20 (5.95, 61.96)	10.98 (2.62, 45.98)
4 + years	%1.%2(1.60, 15.85)	(0.93, 15.60)

^†^ Top quartile 498—4286; 2nd quartile 337—497; 3rd quartile 195—336; 4th quartile 1 – 194

### Virologic outcomes

**Fig. 2:**
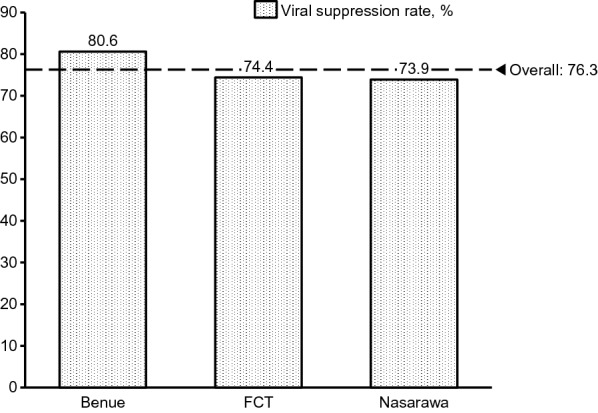
Virologic suppression rates by State

**Fig. 3:**
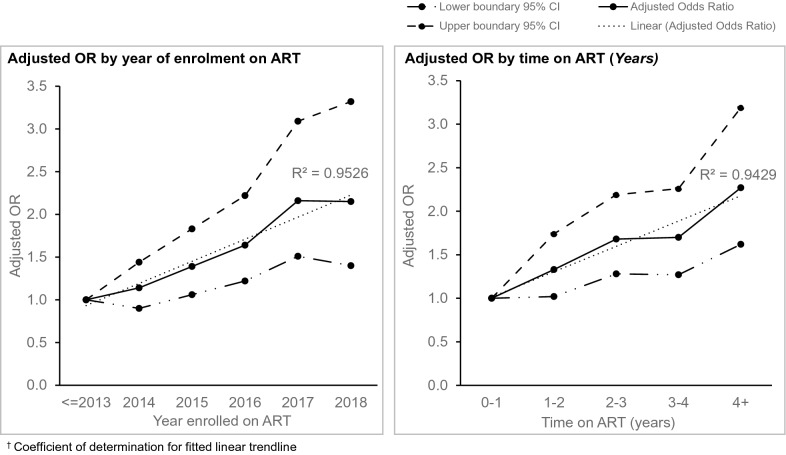
Trends in viral suppression on the private for-profit HIV program

Overall viral suppression rate on the program was 75.6% (95% CI 74.5, 76.4%). As shown in Fig. 2, subjects in Benue had a significantly higher suppression rate of 80.6% (95% CI 78.9, 82.3%), compared to FCT’s 74.4% (95% CI 72.7, 75.9%) and Nasarawa’s 73.9% (95% CI 72.6, 75.2%). Results of univariate and multivariate logistic regressions to determine factors associated with viral suppression are shown in Table 5. In the multivariate model, males were less likely to be virally suppressed compared to females (OR 0.84, 95% CI 0.72–0.98), as were patients in FCT (OR 0.40, 95% CI 0.23–0.69) and Nasarawa (OR 0.40, 95% CI 0.23–0.69) compared to those in Benue state. Patients whose starting regimen was AZT- based were less likely to be virally suppressed compared to those started on TDF- based regimen (OR 0.72, 95% CI 0.61–0.86). As shown in Table 5, decreasing age at ART start was associated with progressively worse viral outcomes, with patients in the bottom age quartile having 29% decreased odds (OR 0.71, 95% CI 0.57–0.88) compared to those in the highest age quartile. Viral outcomes showed significant positive trends over time (ART enrolment year) as well as with increased duration on ART (Fig. 3). Subjects in the lowest quartile of baseline CD4 + had significantly lower odds (OR 0.69, 95% CI 0.57–0.83) of viral suppression compared to those in the highest quartile. Contact testing was associated with increased odds of viral suppression (OR 1.19, 95% CI 1.00–1.41), as was receiving care in model facilities (OR 1.30, 95% CI 1.13–1.50).

**Table 5:** Factors associated with viral suppression in the study population

Variable	Bivariate odds ratio (95% CI)	Multivariate odds ratio (95% CI)
*Sex*		
Female	1.0	1.0
Male	0.86 (0.77, 0.95)	0.84 (0.72, 0.98)
*State*		
Benue	1.0	1.0
FCT	0.70 (0.61, 0.80)	0.40 (0.23, 0.69)
Nasarawa	0.68 (0.60, 0.76)	0.40 (0.23, 0.69)
*Age at ART start*^†^		
1st quartile	1.0	1.0
2nd quartile	0.93 (0.81, 1.07)	0.81 (0.67, 1.00)
3rd quartile	0.93 (0.81, 1.07)	0.79 (0.64, 0.98)
4th quartile	0.88 (0.76, 1.01)	0.71 (0.57, 0.88)
*ART enrollment year*		
2013	1.0	1.0
2014	1.06 (0.90, 1.26)	1.14 (0.90, 1.44)
2015	1.15 (0.98, 1.37)	1.39 (1.06, 1.83)
2016	1.24 (1.07, 1.45)	1.64 (1.22, 2.22)
2017	1.09 (0.92, 1.29)	2.16 (1.51, 3.09)
2018	1.13 (0.94, 1.40)	2.15 (1.40, 3.32)
2019	1.78 (1.16, 2.73)	*
*Duration on ART*		
0–1 year	1.0	1.0
1–2 years	1.01 (0.88, 1.17)	1.33 (1.02, 1.74)
2–3 years	0.99 (0.85, 1.14)	1.68 (1.28, 2.19)
3–4 years	0.97 (0.82, 1.13)	1.70 (1.27, 2.26)
4 + years	0.93 (0.81, 1.08)	2.27 (1.62, 3.19)
*Facility support type*		
Standard	1.0	1.0
Model	0.86 (0.78, 0.95)	1.30 (1.13, 1.50)
*ARV refill duration*		
1 month	1.0	1.0
2 months	1.59 (1.44, 1.76)	1.18 (1.02, 1.36)
3 + months	2.14 (1.82, 2.52)	1.94 (1.54, 2.43)
*Baseline CD4*^‡^		
1^st^ quartile	1.0	1.0
2nd quartile	1.01 (0.86, 1.20)	1.07 (0.87, 1.30)
3rd quartile	0.97 (0.82, 1.14)	1.03 (0.85, 1.25)
4th quartile	0.63 (0.54, 0.74)	0.69 (0.57, 0.83)
*ART regimen at start*		
TDF based	1.0	1.0
AZT based	0.72 (0.64, 0.81)	0.72 (0.61, 0.86)
Other	*	0.88 (0.31, 2.50)
*Contact tested*		
No	1.0	1.0
Yes	1.16 (1.01, 1.33)	1.19 (1.00, 1.41)

^†^ 1st quartile ≥ 41 years; 2nd quartile 33—40 years; 3rd quartile 28—32 years; 4th quartile 18—27 years

^‡^ Top quartile 498—4286; 2nd quartile 337—497; 3rd quartile 195—336; 4th quartile 1 – 194

^*^ Variable dropped in model due to too few observations

## Discussion

Few reports on patient level HIV treatment outcomes from Nigeria have been published, and there is no large-scale outcomes data from private for-profit health facilities. The potential to improve equity and access to health services in low and middle income countries (LMICs) through better private sector engagement is widely recognized in the literature, yet very little empirical information on their effectiveness and quality of service exists [16, 17, 19, 28–33]. This study provides the first private sector data on patient outcomes in a large-scale HIV treatment program in Nigeria. Benue, Nasarawa and FCT are all in the north-central zone of the country, with significant HIV burdens—5.3%, 2.0% and 1.6% respectively, compared to a national average of 1.7% [1].

Population estimates for viral suppression among PLHIV in Nigeria vary widely between geo-political zones, ranging from 33.7% in the south-south zone to 65.7% in the north-central [1]. We found an overall viral suppression rate of 75.6% in our study, strengthening the case for expanding the role of private for-profit facilities in HIV care and treatment. Although the viral suppression rate compares favorably with other available data, it falls short of the last 95 of the UNAIDS 95-95-95 targets, pointing to the need to intensify programmatic support and capacity building for these facilities to enhance their quality of care and patient outcomes. This study found better retention in care, lower mortality and higher viral suppression rates in Benue, likely attributable to a longer history of large-scale HIV care and treatment services in the state, as it initially was the target of much programming support, being the first epicenter of Nigeria’s HIV epidemic.

Females had better outcomes, including retention in care and viral suppression, which is in keeping with other studies from Nigeria and elsewhere [34–37]. Better health seeking behavior of women and earlier detection through Prevention of Mother To Child Transmission (PMTCT) interventions may contribute to the observed better outcomes. Low baseline CD4 + counts, use of AZT-based regimens and shorter duration on ART were associated with worse virologic outcomes in this study, consistent with findings of other researchers in Africa and elsewhere [38–45]. This observation was because model facilities received a higher level of attention from program Quality Improvement (QI) teams, who identified patients failing, or at risk of failing treatment sooner and made the necessary switches.

Despite the potential that private for-profit health facilities hold, engaging them in public and social services like HIV prevention, care and treatment services is fraught with challenges [17, 28–30, 32]. Private providers are poorly regulated with unclear standards of care that often translates into variable quality of care [28]. In many countries, effective engagement, oversight and regulation of the various private healthcare providers may be constrained by imperfect strategic intelligence, limited financial influence and weak institutional capacity [30]. Private facilities are generally small in size, which, coupled with the financial burden associated with private healthcare, results in fewer patients per facility, making program support less efficient [29]. In addition, the core business model of for-profit by the private sector may limit transparency in operations and impose user fees which often makes it difficult for donors to reconcile their goals of minimizing out of pocket expenses [17, 28]. The facilities included in this study generally face many of these challenges.

Nonetheless, our study showed that intensified technical support for these facilities (model facility support) yielded significant improvements in quality of care and patient outcomes. Furthermore, performance improved with experience, and outcomes have continued on an upward trajectory (Fig. 3). The scale, reach and acceptability of private health facilities make them especially critical to achieving UNAIDS 95-95-95 by 2030 targets.

This study was limited by the non-availability of some data for analysis. Trends in repeat viral load and CD4+ testing records could be informative in understanding determinants of disease progression in the study population but repeat test measures were unavailable for analysis. Also, because viral load testing was not universally accessible during the earlier years of program implementation, testing was prioritized for patients in whom it was specifically indicated, such as those with signs of clinical failure or evidence of poor adherence. This may have resulted in an under-estimate of viral suppression rates and may affect the overall generalizability of our findings.

## Conclusion

Private for-profit facilities are a major provider of HIV and other health services in Nigeria and other LMICs. Our study shows that despite limited support, they are able to provide quality care for PLHIV. With appropriate technical support and engagement, private for-profit health facilities can help accelerate the achievement of UNAIDS 95-95-95 target by 2030.

## Data Availability

The datasets generated and analyzed for this study are not publicly available due to ethical reasons, but can be accessed from the corresponding author upon reasonable request.

## Abbreviations

NAIIS: Nigerian National AIDS Indicator and Impact Survey
PHIA: Population-based Impact Assessment
PLHIV: People Living with HIV
ART: Antiretroviral Treatment
SCIDaR: Solina Center for International Development and Research
IHVN: Institute of Human Virology, Nigeria
FCT: Federal Capital Territory
PEPFAR: President’s Emergency Plan for AIDS Relief
RADET: Retention and Audit Determination Tool
LTFU: Loss to follow up
